# Transcriptional activation of candidate paused genes after partial hepatectomy

**DOI:** 10.17912/micropub.biology.001160

**Published:** 2024-06-10

**Authors:** Franck Mayeux, Nicolas Buisine, Olivier Fayol, Patricia Uguen

**Affiliations:** 1 UMR-S1174, Université Paris-Saclay, Orsay, France; 2 UMR CNRS 7221, Muséum national d'Histoire naturelle, Paris, France; 3 UMR 3348, INSERM U1278, Institut Curie, Université Paris-Saclay, Orsay, France

## Abstract

Partial hepatectomy is a model of acute liver injury that is known to induce a strong reprogrammation of gene expression. Transcriptional induction of Immediate Early Genes is extremely fast and this would be due to the release of RNA Polymerase II poised for elongation at ‘paused’ genes. Using bioinformatic analysis, we identified 23 genes sharing features of paused genes before hepatectomy, and with predicted quick and strong expression induction after. This transcriptional dynamic, confirmed by RT-qPCR for *Jun* , *Fos* , *Btg2,* is very precocious. RNA Pol II CTD Ser2 hyperphosphorylation indicates a switch to productive elongation and release from transcriptional pause.

**Figure 1. Activation of RNA Pol II precedes over-expression of candidate paused genes after partial hepatectomy:**
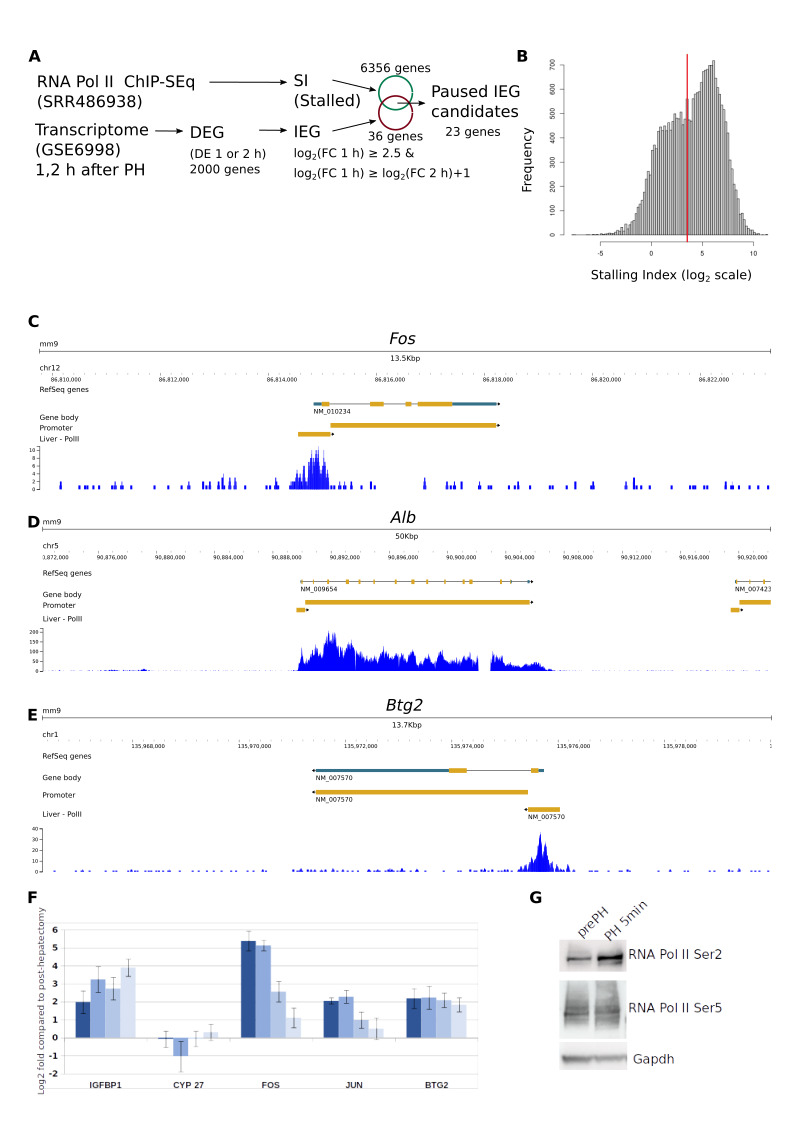
(A) Experimental workflow. (B) Frequency of gene stalling index (SI). The red line indicates the stringent cut-off for RNA Pol II enrichment at the 5’ proximal region of the genes. (C) Example of candidate paused gene: Fos. (D) Example of constitutively expressed genes, without evidence of RNA Pol II enrichment at the 5’ proximal region: Alb. (E) RNA Pol II enrichment at the 5’ proximal region of the paused candidate Btg2 gene. (F) Quantification by RT-qPCR of gene expression following PH *in vivo* : time intervals (from deep to light blue) are 15 min, 30 min, one and two hours. (G) Western blot of RNA Pol II phosphorylation before (prePH) and 5 min after PH (PH 5min).

## Description

Liver is the central organ safeguarding metabolic homeostasis in mammals. It not only assures biosynthesis and storage of various nutrients, glucids, fats and vitamins, it is also essential for the detoxification of xenobiotic substances (see Taub 2004). The liver has the capability to restore its initial mass after injury or partial hepatectomy (PH) while still maintaining its function. Liver injury (such as two-thirds hepatectomy) is immediately followed by the entry in the first step of a regenerative process. In this step called “priming”, hepatocytes exit from a quiescent state (G0) and enter into G1 phase for 4 hours. This coincides with the activation of early immediate genes (IEG) such *Atf3* , *C-Jun* or *C-Fos* for which transcriptional regulation is independent of protein synthesis (Webber et al. 1995; Zimmermann 2004) . Hepatocytes start to actively divide 36 hours after PH, and regeneration is finally reached by 7 to 10 days, under the control of cell cycle genes (see Taub 2004).

RNA Pol II is often stalled at the 5' proximal region of genes (5PRG) in 20 to 40% of the cases (Guenther et al. 2007; Muse et al. 2007; Zeitlinger et al. 2007; Core and Lis 2008) . This post-transcriptional regulation is key to synchronize gene expression changes in response to stress or for appropriate development (Gaertner and Zeitlinger 2014) . This non-productive state is released after phosphorylation by the catalytic subunit of the P-TEFb complex (CDK9) of the Ser2 residues located on the C-terminal domain (CTD) of RNA Pol II, together with the Negative Elongation Factor-E (NELF-E) and 5,6-dichloro-1-b-D-ribofuranosylbenzimidazole sensitivity-inducing factor (DSIF) regulators of elongation (Fujinaga et al. 2004) . In this paper, we hypothesize that IEG transcription is poised for elongation (paused) in normal context and released after a stress such as hepatectomy. We ask whether a link can be drawn between RNA Pol II enrichment at the 5PRG and emergency transcriptional induction following PH. We found 23 IEG sharing characteristic features of paused genes and collected evidence of a fast transcriptional induction following phosphorylation of Ser2 at CTD of RNA Pol II.

The point is to identify the key IEG specifically released from transcriptional pause after PH. To this end, we used a bioinformatic-based comparison of two datasets: one corresponding to a RNA Pol II ChIP-Seq carried out in wild type and untreated mouse liver (Sun et al. 2011) , and a micro-array-based time course measure of gene expression changes following PH (Otu et al. 2007) . Our rationale is to predict 'paused' genes based on ChIP-Seq reads density distribution along genes on the one hand, and IEG from micro-arrays data on the other hand. IEG released from transcriptional pause should score in both gene lists ( Figure 1A ).

RNA Pol II density can be estimated from ChIP-Seq reads density along genes (Guenther et al. 2007; Muse et al. 2007; Zeitlinger et al. 2007; Core and Lis 2008) , where a key feature of paused genes corresponds to a strong accumulation at the 5' end, and a much fainter (or no signal) signal spread along the gene body (GB). To this end, we counted the total number of reads in a 600 bp sliding windows within a 5kb region centred at the predicted Transcriptional Start Site (TSS) of all genes and kept the highest value as the estimate of RNA Pol II density at promoters ( Figure 1B ). We used the median value of reads count in non overlapping 600 bp windows along the rest of the gene as the estimate of RNA Pol II density in GB. We computed a Stalling Index (SI) as the ratio of the highest read count at promoter region over the median accross GB (therefore making the ratio independent of gene size). With this set up, the highest the SI, the stronger the contrast between promoter and GB read density. SI ranges from -5 to > 10 (in log _2_ scale) and follows a bimodal distribution with a local minima at SI ≈ 3.6 ( Figure 1B ). This corresponds to 1942 genes, highly enriched in GO terms predominantly related to numerous biosynthetic processes and cell cycle, fitting well with the know physiological metabolic activity of the liver (Webber et al. 1995; Zimmermann 2004) . Our approach readily identified gene known to be functionally 'paused' in other tissue, namely *Fos* , *Jun,* *Myc* (extended data Table 1) (Fujita et al. 2008) . *Fos* is the school case example of paused genes, with strong enrichment at 5PRG and background-level signal over the GB ( Figure 1C ), strongly suggesting it is also paused in liver. In contrast, Albumine ( *Alb* ) is the archetype of liver-specific genes with very strong and constitutive transcription, and for which no RNA Pol II accumulation is expected at the 5PRG. Indeed, we found a strong accumulation of RNA Pol II all along the GB with limited contrast relative to the 5PRG ( Figure 1D ).

A relatively large number of differentially expressed genes (1506 and 1763 after 1 and 2 hours, respectively, see Micro-array data processing section) are induced by PH (Otu et al. 2007) . A total of 1057 genes have log _2_ (FC 1 hour) ≥ 1.5, and 1615 genes with log _2_ (FC 2 hour) ≥ 1.5, equating to a non-redundant set of 2000 DE genes. This result, well in agreement with previous analysis (Otu et al. 2007) , shows that gene transcription is strongly altered quickly after PH.

We first used a stringent set of parameters to isolate paused genes candidates. We first restrict our choice to a strong induction at 1 hour post PH but not at 2 hours. This translates into log _2_ (FC 1 hour) ≥ 2.5 and log _2_ (FC 1 hour) ≥ log _2_ (FC 2 hour) +1, i.e. a log _2_ (1) = 2 fold difference. The second parameter is SI ≥ 3.7 ( Figure 1A ). This identifies a set of seven genes: *Gadd45b* , *Ddit4* , *Atf3* , *Tomil2* , *Ect2* , *Fos* , *Btg2* (extended data Table 1). Using more relaxed parameters (SI ≥ 2.5) results in an extended list of 23 genes, including the known paused genes *Jun* (SI=3.03) and *Myc* (SI=2.51). Examples of non-candidate genes are shown in extended data Figure 1 : *Actb* is a gene with high SI but not being DE, *Igfbp1* is a DE gene but with low SI and *Cyp27b1* is a gene with low SI and not being DE. Our seven candidates are transcription factors and cytoplasmic factors acting on the control of cell cycle, response to DNA damage and intracellular trafficking. We selected *Btg2* for further experimental validation ( Figure 1E ) since it has been already described being overexpressed after PH (Zhang et al. 2009) .

We next followed the expression changes of a few selected genes by RT-qPCR ( Figure 1F ): *Igfbp1* is a positive control with expression increasing over time after PH; *Cyp27a* should display a low level of expression after PH; the two known IEG but with unknown paused status *Fos* and *Jun* in liver; and one of our candidates, *Btg2* . As expected, *Igfbp1* expression steadily increases, while *Cyp27a* is not over-expressed as expected (Su et al. 2002; Otu et al. 2007) . We could confirm that *Fos* and *Jun* expression is induced very early PH but only transiently. *Btg2* expression is strongly induced as early as 15 min and slowly decreases over time. Altogether, gene expression changes agree well with micro-array data, therefore supporting the idea of transcriptional induction resulting from pause released.

It is well established that RNA Pol II processivity is dictated by the phosphorylation of Ser2 and Ser5 at the CTD (see Price 2000): release from transcriptional pause and productive elongation correlate with Ser2 phosphorylation. We thus monitored the phosphorylation status of RNA Pol II by Western blot with anti phospho-Serine-2 and anti phospho-Serine-5 antibodies ( Figure 1G ). We found a clear enhancement of the ratio of Ser2 over Ser5 phosphorylation as early as five min post PH, thereby revealing an extremely fast entry of the RNA Pol II complex into productive elongation.

Based on these evidences, we would like to propose that *Jun* , *Fos* and *Btg2* , together with the other genes (extended data Table 1), are candidate genes poised for elongation and therefore correspond to paused genes in liver. Their status as IEG has been the subject of previous work, but the mechanistic details of their transcriptional regulation haven't been addressed so far. Our work is a first step toward understanding the connection between pause release and liver emergency responses.

## Methods

***Surgical procedure***

C57Bl/6J mice were originally ordered from Janvier Labs (Le Genest-Saint-Isle, France) and raised under standard husbandry protocols in our facility. A backcross with C57Bl/6J from Janvier Labs were performed every 2 years. All animals received humane care according to the criteria outlined in the “Guide for the Care and Use of Laboratory Animals” written by the National Academy of Sciences and published by the National Institutes of Health. The experiments were approved by the ethical committee C2EA59 and by the French ministry of research. Two-thirds hepatectomy (PH) were performed on male mice aged of 12 to 16 weeks, as described, with removal of the left and right lateral lobes (Mitchell and Willenbring 2008) . Surgery and samples harvesting were performed under general anaesthesia induced by isoflurane inhalation (2%, 0.8L/min) on fed mice. At different times after PH (15 min, 30 min, 1 hour and 2 hours), mice were euthanized by isoflurane inhalation (4%) and liver pieces were immediately frozen in nitrogen-cooled isopentane or stored in RNA *later* buffer (Qiagen) and stored at -80°C until use.

***Quantitative RT-PCR***

RNA (1μg) was extracted from mouse liver with Tri reagent (Sigma) as recommended by manufacturer. Reverse transcription was realised by superscript II enzyme and random primers (Invitrogen). Quantification of cDNAs was carried out with the SYBR green PCR kit (Bio-Rad) on a Chromo 4 Real-Time detector (Bio-Rad). Detections were realised at least as biological duplicates and relative transcription levels were normalized over the housekeeping gene GAK. Data processing was performed with the Opticon Monitor 3 software (Bio-Rad) using 2 ^-DDCT^ method (Livak and Schmittgen 2001) . Seven mice were analysed for Pre-PH condition and for 30 min, 1 and 2 hours time points after PH, and 4 mice were tested for 15 min after PH.

***Western blot analysis***

Liver tissue was ground with potter in buffer L (HEPES 10mM, EDTA 0,2 mM, KCl 10 mM, MgCl _2_ 1,5 mM, NaCl 150 mM, 0,2 % NP40 and DTT 1 mM and 40 Units RNAse OUT). Lysates were clarified by centrifugation at 500g for 5 min, then the supernatant is centrifuged at 9000g for 5 min to recover the supernatant. Proteins were quantified by Bradford. Protein extracts were sonicated for 5 min at high intensity, 0.5 Off and 1 On on a Bioruptor apparatus. Lysates were separated on 10% SDS-PAGE and transferred to nitrocellulose membranes. Blots were incubated with antibodies against RNA Pol II Ser5 (Abcam ab5131), RNA Pol II Ser2 (Abcam ab5095), GAPDH (Abcam, ab8245), then detected by anti-IgG-HRP with ECL (Bio-Rad).

***ChIP-Seq data processing***

RNA Pol II ChIP-Seq reads from untreated mouse liver were extracted from the SRR486938 dataset at SRA (Sun et al. 2011) . After quality control with FastQC, reads were mapped on Mm9 genome assembly available at UCSC with BOWTIE (Quinlan and Hall 2010) using stringent parameters (-q -l 26 -n 3) and piped to a simple AWK script to convert to BED file format. Reads density was computed with the genomeCoverageBed software from the BEDTOOLS package v2.16 (Quinlan and Hall 2010) and stored in BEDGRAPH format (-bg).

Mm9 annotation (UCSC) was used to derive the coordinates of the 5PRG defined as a 600 bp window centered at the predicted TSS. This corresponds to the region where stalled RNA Pol II presence is expected to be high. The rest of gene models were used to define 'gene body' (GB). Reads count within 5PRG and GB was computed with intersectBed from BEDTOOLS.

Visualization of genome wide profiles and annotations were performed with the JBROWSE genome browser (Buels et al. 2016) after conversion to BIGWIG format.

***Micro-array data processing***

Liver transcriptome changes after partial hepatectomy were characterized by micro-array analysis of RNA samples collected over relatively short time points (1 and 2 hours) in order to capture IEG (Otu et al. 2007) . Micro-array data were processed within the R environment. Signal was normalized with GCRMA and differential analysis was performed with the LIMA package (Lim et al. 2007) . Data were downloaded from GEO as GSE6998. Differentially expressed genes were defined with a relatively strong threshold (| log _2_ (Fold Change) | ≥ 2). Gene Ontology analysis was performed with the GhOST API of the gProfiler website (Zhang et al. 2009) using all mouse gene names as a background.

## Reagents

***Sequence of primers used for RT-qPCR***

**Table:** 

**Gene**	**Sequence (5’ to 3’)**
*C-Jun*	Fw : GGCTAACCCCGCGTGAA
*C-Jun*	Rv : AAGGTCGTTTCCATCTTTGCA
*C-Fos*	Fw : GGGACAGCCTTTCCTACTACC
*C-Fos*	Rv : GATCTGCGCAAAAGTCCTGT
*Cyp27a1*	Fw : CCTCACCTATGGGATCTTCATC
*Cyp27a1*	Rv : TTTAAGGCATCCGTGTAGAGC
*Igfbp1*	Fw : ATCAGCCCATCCTGTGGAAC
*Igfbp1*	Rv : TGCAGCTAATCTCTCTAGCACTT
*Gak*	Fw : CTGCCCCACCAGGCATTTG
*Gak*	Rv : CCATGTCACATACATATTCAATGTACCT
*Btg2*	Fw : GGCTATCGCTGTATCCGTATCA
*Btg2*	Rv : TGCGGTAAGACACTTCATAGGG

## Extended Data

Description: List of candidate genes having a SI higher than 2.5 and having a strong induction of expression 1 hour after partial hepatectomy. Resource Type: Dataset. DOI: 10.22002/xqcn3-12317

Description: RNA polymerase II enrichment at the 5' proximal region of non candidate genes. Resource Type: Image. DOI: 10.22002/mctvw-4yx47
